# Cross-neutralization of Influenza A by SARS-CoV-2 specific neutralizing antibodies and polyclonal plasma: Is pre-exposure to SARS-CoV-2 protective against Influenza A?

**DOI:** 10.1016/j.heliyon.2024.e40638

**Published:** 2024-11-22

**Authors:** Mohammad Mamun Alam, Asma Salauddin, Sayra Moni, Md Belayet Hasan Limon, Raisha Musarrat, Sagar Bosu, Mohammad Enayet Hossain, Mohammed Ziaur Rahman, Mustafizur Rahman

**Affiliations:** Virology Laboratory, Infectious Diseases Division, International Centre for Diarrhoeal Disease Research, (icddr,b), Bangladesh

**Keywords:** Cross-protection, Influenza type A, SARS-CoV-2, Neutralizing antibody

## Abstract

According to sparse information from various countries, the seasonal influenza virus circulation has drastically decreased during the COVID-19 pandemic. Here, we show the cross-reactivity of anti-SARS-CoV-2 antibodies against influenza viruses. Plasma samples were collected from 311 SARS-CoV-2 infected individuals. The samples were tested for antibody titers against SARS-CoV-2 by ELISA and seasonal influenza virus strains (influenza A/H1N1, A/H3N2, B/Yamagata, and B/Victoria) using a Hemagglutination Inhibition Assay (HAI). In addition, SARS-CoV-2 antibody-positive but Influenza antibody-negative samples (n = 16) were investigated to determine the SARS-CoV-2 antibody-neutralizing potential against influenza viruses by microneutralization (MN) assay. The SARS-CoV-2 genomes were sequenced using Illumina next-generation sequencing, and an in-silico protein structural analysis was performed to identify epitope and antibody binding similarities between SARS-CoV-2 and influenza viruses. Among 16 samples that didn't contain antibodies against Influenza A strains (H1N1 and H3N2), five showed high (MN titer≥20), and six showed moderate (MN titer≥10) capability to neutralize Influenza A. Subsequent in-silico analysis revealed that most efficient binding (>8 kcal/mol) was found between the antibodies of SARS-CoV-2 delta variant (ΔG) with influenza A/H1N1 HA (Hemagglutinin), A/H3N2 HA, A/H1N1 NA (Neuraminidase), and A/H3N2 NA glycoproteins with −12.4, −9.3, −10.1, and −11.7 kcal/mol, respectively. This investigation revealed that neutralizing antibodies of the delta variant cross-reacted with the Influenza A virus, which might protect against influenza viruses and reduce and shift the seasonal influenza circulation during the COVID-19 pandemic. Our findings warrant further study to explain the probable mechanisms of this cross-reactivity.

## 1 Introduction

The distinguishing characteristics of influenza viruses are their segmented, negative-strand RNA genomes. The ability of the influenza virus to undergo antigenic drift and shift is facilitated by the distinctive structure of its genome and the activity of its viral proteins. Consequently, viruses have the ability to render the efficacy of the host's enduring adaptive immune responses. Seasonal influenza epidemics are observed in temperate parts of the northern and southern hemispheres throughout the months of November to March in the northern hemisphere and April to September in the southern hemisphere, respectively [1,2]. Bangladesh, a tropical nation situated in the northern and eastern hemispheres, experiences an annual seasonal influenza outbreak that normally occurs during the monsoon season, spanning from May to September [3]. In contrast to previous seasons, the 2019-20 influenza season concluded unusually early in China [4], and there was a significant decrease in influenza circulation in the United States and several Asian countries [[5], [6], [7], [8]] in the Northern Hemisphere, including Bangladesh [9]. Similar events were also documented in the Southern Hemisphere. There are other variables contributing to the decline in influenza virus activity, such as the global pandemic caused by SARS-CoV-2 and the implementation of public health measures aimed at its prevention [[9], [10], [11]]. Similar clinical symptoms, such as fever, cough, headache, muscle and joint pain, severe malaise, sore throat, runny nose, anosmia, and ageusia, are caused by both influenza and SARS-CoV-2 [12]. The transmission of SARS-CoV-2 and influenza viruses occurs via aerosolized or respiratory droplets in interpersonal contact [13]. Since the emergence of the COVID-19 pandemic, scholars have hypothesized the potential occurrence of coinfection involving both viral agents [12]. Moreover, it has been shown that there were occurrences of coinfections in the initial stages of the pandemic [14]. Yue et al. [15] reported a notable prevalence of coinfection between SARS-CoV-2 and influenza viruses, with influenza A accounting for 49.8 % and influenza B accounting for 7.5 % of the cases. The initial identification of three laboratory-confirmed cases of SARS-CoV-2 occurred on March 8, 2020, coinciding with a significant prevalence of influenza in Bangladesh. Since then, the SARS-CoV-2 virus has continued to circulate. Despite the observed influence of COVID-19 transmission on the prevalence of other infectious diseases, there is currently a lack of study examining the specific effects of COVID-19 on the incidence of influenza. The decline in influenza activity suggests that the implementation of COVID-19 protocols has effectively mitigated the transmission of further viral respiratory illnesses.

The SARS-CoV-2 virus surfaced in the latter part of 2019 and swiftly disseminated across several nations, resulting in the infection of millions of individuals and precipitating a worldwide COVID-19 pandemic [16]. The trimeric spike glycoprotein (S) of SARS-CoV-2 interacts with angiotensin-converting enzyme 2 (ACE2), resulting in the fusion and entry of the virus into host cells [17,18]. Viral fusion machineries of Type 1, such as Influenza hemagglutinin (HA), HIV-1 Env, and SARS-CoV-2 S protein, facilitate the entrance of viruses through structural rearrangements. These fusion machineries exist as trimers in both their pre-fusion and post-fusion states [[18], [19], [20], [21]]. The S protein of SARS-CoV-2 is enveloped by glycans generated from the host organism, with each trimer containing such glycans. Through site-specific glycan analysis, it has been determined that around 28 % of the glycans present on the protein surface are oligo mannose-type glycans that have undergone incomplete processing [22]. Enveloped viruses, such as SARS-CoV-2 and influenza, frequently exploit the glycosylation pathways of host cells, hence exerting an impact on pathobiology and immune selection [23]. The presence of these glycan patterns has the potential to facilitate immune evasion or viral neutralization through the generation of cross-reactive antibodies, which in turn may result in antibody-dependent enhancement (ADE) of infection [23]. In this study, we investigated the clinical characteristics and outcomes associated with infections caused by SARS-CoV-2, influenza virus, and the simultaneous presence of both viruses during the COVID-19 pandemic in 2020. Furthermore, our study examined the potential cross-protective effects of SARS CoV-2 antibodies against Influenza.

In this study, we aimed to demonstrate that the distinctive characteristics of SARS-CoV-2 spike protein broadly neutralizing antibodies (bnAbs) enable them to effectively identify and assimilate viral glycans. The Influenza HA protein glycan shield can be targeted by SARS-CoV-2 spike protein-specific bnAbs. We also described the in silico prediction of protein-protein interaction between SARS-CoV-2 spike protein-specific bnAbs and influenza epitops.

## 2 Materials and methods

### 2.1 Ethics statement

This study, conducted by the International Centre for Diarrhoeal Disease Research (icddr,b), Bangladesh has received approval from the institutional review board, specifically the Research Review Committee and Ethical Review Committee (protocol no. PR-21065). The patients were provided with written informed consent, and they were assured that their personal information, including their names and identify, would not be disclosed. The collected data will be utilized to enhance patient care activities, including the dissemination of findings through publishing.

### 2.2 Study Design and participant

In the present investigation, a total of 311 blood samples were obtained from infected individuals from patients on days 90 following the diagnosed with SARS-CoV-2 infection and underwent COVID-19 testing at the icddr,b facility. These plasma samples were accompanied by the corresponding medical records of the participants. Data on demography, monthly income, and history of past SARS-CoV-2 infection were collected using a standardized questionnaire. Furthermore, the participants' blood samples, measuring 4 mL, were obtained upon enrolment. The diagnostic procedure involved the utilization of nasopharyngeal swabs, which were subjected to reverse transcription polymerase chain reaction (RT-PCR) testing in order to detect the presence of SARS-CoV-2. The RT-PCR assay was conducted in accordance with the standard set by the World Health Organization (WHO), which focuses on the envelope E, N, and RdRp genes of the SARS-CoV-2 virus [24].

### 2.3 Specimen processing

The plasma was isolated from the blood using centrifugation at a force of 400 times the acceleration due to gravity (400×*g*) and afterwards preserved at a temperature of −80 °C within a freezer. The isolation of peripheral blood mononuclear cells (PBMCs) was conducted using the process of density gradient centrifugation with a force of 500 times the acceleration due to gravity (500×*g*) utilizing Ficoll–Hypaque. The PBMCs that had been isolated were subjected to a washing step and subsequently cryopreserved in a solution containing 10 % dimethyl sulfoxide (DMSO) in fetal bovine serum (FBS). The cryopreserved cells were stored in liquid nitrogen until they were ready for further experimentation [25].

### 2.4 Hemagglutination Inhibition Assay (HAI)

The HAI assays were conducted in order to identify Influenza-specific neutralizing antibodies in plasma samples, following the methodology outlined in a recent study [26]. The prototype antigen strains for influenza A were A/Kansas/14/2017(H3N2) (FR-1666) and A/Brisbane/02/2018 (H1N1) (FR-1665), respectively. The B/Phuket/3073/2013_YAM (FR-1735), and B/Washington 02/2019_VIC (FR-1733) antigen strain (provided by IRR) was employed in the context of the influenza B virus. In summary, a total of 311 plasma samples were subjected to an overnight treatment with a receptor-destroying enzyme and afterwards heat-inactivated to mitigate any potential nonspecific inhibitory effects. Additionally, the sera were subjected to an adsorption process using red blood cells to eliminate any nonspecific agglutinins. Serial dilutions were performed in a two-fold manner, starting with an initial dilution of 1:8 in a microtiter plate containing negative control and positive control (reference sera (FR-1683, FR-1682, FR-1738 and FR-1685 for H3N2, H1N1, VIC and YAM respectively provided in the kit). Following this, the plates were incubated with prototype antigen strains at standardized concentrations of 4 HA units per 25 μL. Red blood cells from turkeys were introduced into the wells and permitted to undergo sedimentation. The HAI antibody titers specific to each strain were determined for each subject at each time point. For each sera, we had replicated the process three times.

### 2.5 Assessment of SARS-CoV-2-specific antibodies

ELISA experiments were conducted in order to detect SARS-CoV-2-specific IgG and IgM antibodies that are specific to S antigens [27]. In this study, 96-well high-binding microtiter plates (Corning Costar no. 3361) were utilized. The plates were coated with 100 μL of S recombinant protein, with a concentration of 2 μg/mL, in the Coating buffer (CB). The coating process was carried out overnight at a temperature of 4 °C. On the subsequent day, the wells were subjected to a 2-h blockage period at room temperature (RT) using 100 μL of a 0.5 % solution of skim milk (SM) in PBST (PBS supplemented with 0.05 % Tween 20). Subsequently, the plates underwent a washing process, followed by the addition of plasma samples in a volume of 100 μL. These samples were pre-diluted at a ratio of 1:400 in PBST containing 0.5 % SM. The plates were then incubated for a duration of 1 h at a temperature of 37 °C. Following the washing step, the secondary conjugated antibody was introduced at a dilution of 1:1000 in PBST containing 0.5 % SM. The mixture was then incubated for a duration of 1 h at a temperature of 37 °C. The detection of specific IgG antibodies was accomplished using anti-human HRP conjugates, namely the Anti-IgG1 Rabbit Polyclonal Antibody (HRP) from Sino Biological (10702-T16-H). The plates underwent a second washing process, followed by the addition of 100 μL of the substrate OPD (o-Phenylenediamine dihydrochloride chromogenic substrate, Super Sensitive, Sigma). The mixture was then incubated for a duration of 15 min. The reaction was terminated by the addition of 100 μL of 0.5M H₂SO₄ and promptly measured at a wavelength of 450 nm using a spectrophotometer (SynergyHTX, BioTek). The cut-off value was taken as the mean + 3 SD of negative control sera. Thus, an OD value above this was considered positive [28]. We also performed the quantative ELISA of 16 specific samples to determine their Ab titre, we used three controls (High, Standard and low controls) during this process.

### 2.6 Pseudo virus neutralization assay (PNA)

The determination of the neutralizing capacity of plasmaantibodies against SARS-CoV-2 was conducted by the utilization of a pseudovirus neutralization assay (PNA) [29]. The experimental procedure for the SARS-CoV-2 pseudotyped viral neutralization test involved the initial preparation of serial dilutions of the samples in triplicate under investigation. These diluted samples were subsequently combined with a certain quantity (1000 TCID_50_/ml) of the pseudotyped virus. Subsequently, the target cells underwent a 24-h incubation period, during which the quantification of pseudotyped virus entry into the target cells was determined by assessing luciferase expression. This measurement allowed for the determination of the neutralizing antibody concentration within the samples. The NT_50_, which represents the half-maximal neutralization titer, was computed **(**Supplementary Figure-1**)**.

### 2.7 Micro neutralization (MN) assay

MN assay was performed to determine whether a plasma sample contained antibodies that block influenza A virus infection [30,31]. Prior to the implementation of the MN assay, the determination of the 50 % tissue culture infectious dose (TCID_50_) for the influenza virus was conducted using the Reed-Muench method [32]. In this study, the sera obtained from the patients were subjected to heat inactivation. Subsequently, serial 2-fold dilutions were performed on each set of sera triplets, starting with an initial dilution of 1:10. These diluted sera were then incubated with the Influenza A (H1N1) virus strain (100 TCID_50_/ml) in 96-well microtiter plates that contains controls (negative controls and positive controls that contain high titre of Ab against influenza) **(**Supplementary Figure-2**)**. After conducting a response time, Madin Darby canine kidney (MDCK) cells in the log-phase of development were introduced into the wells. The plates were thereafter placed in an incubator and kept at a temperature of 37 °C with a carbon dioxide concentration of 5 % for a duration of 20 ± 2 h. The measurement of antibody responses against the influenza virus was conducted through the utilization of mouse anti-influenza virus labeling Ig G (H + L) secondary antiboy. In this study, a monoclonal antibody targeting nucleoprotein (NP) was utilized as the primary staining antibody. Additionally, a secondary staining antibody, specifically goat anti-mouse IgG horseradish peroxidase (HRP) conjugated, was employed. The absorbance values were measured utilizing a spectrophotometer, and afterwards, the 50 % viral neutralization (NT_50_) titer of each plasma was determined. The titers of neutralizing antibodies were determined as the reciprocal of the greatest dilution of plasma samples that exhibited a minimum of 50 % neutralization. Plasma MN titer≥1:10 was used as a cut-off for indicating past infection [33].

### 2.8 Sequencing

The nCoV-2019 sequencing methodology v3 (LoCost) [34] was followed in the preparation of the sequencing libraries. In short, before being combined for purification and sequencing adapter ligation, a batch of 24 amplified samples was barcoded. After quantification, the resulting libraries were injected onto an Oxford Nanopore MinION MK 1C platform for 8 h using a FLO-MIN106D (R9.4.1) flow cell. A total of 2,890,603 reads (average length, 499 bps) with a range of 71,821 to 170,241 reads per sample were generated by real-time base calling with Guppy 4.3.4, which was made available with MinKNOW software in rapid base-calling mode. Based on ARTIC Field Bioinformatics software (https://github.com/artic-network/fieldbioinformatics), a consensus FASTA file was created utilizing a FASTQ QC + ARTIC + Nextclade r1.0.4 workflow on the cloud-based analytic platform EPI2ME Desktop Agent v3.3.0 with default parameters.

### 2.9 Protein structure analysis

Our hypothesis suggests that the potential for cross-reactivity between SARS-CoV-2 and the influenza virus may arise due to structural similarities observed in the outer proteins of these two viral entities. The presence of such similarities may give rise to the possibility of cross-reactivity if the epitopes present on the viral envelope become exposed to the immune system, thereby eliciting the proliferation of analogous antibodies. The TM-Align tool developed by Zhang Lab was employed for conducting sequence-independent comparisons of protein structures [35]. The TM score is a numerical scale that spans from 0 to 1, where a value of 1 signifies optimal alignment. In our study, we utilized a TM-score range of 0.0–0.30 to indicate a stochastic resemblance in protein structures, whereas a score between 0.5 and 1.0 was regarded as indicative of proteins exhibiting a common fold. The ClusPro server [36], a well-established tool for protein-protein docking, was utilized in our investigation to perform docking of Hemagglutinin (HA) and Neuraminidase (NA) proteins with the wild type and delta variant SARS-CoV-2 spike protein antibodies (PDB ID: 7YCK and 7W9E respectively). During the course of this experiment, we employed the Antibody mode of the server to analyze the proteins by considering them as ligands and the antibody as the receptor [37]. The initial step involved selecting the top-ranked model from a set of complex models based on its significant cluster size and highest weighted score. Subsequently, in order to assess the strength of interaction, we conducted a detailed analysis of this complex using the Prodigy server. This analysis involved calculating the binding energy between the antibody and the protein, with careful consideration given to specifying the chain numbers for both molecules during input. By employing this approach, we were able to gain a comprehensive understanding of the docking and binding properties exhibited by these biological molecules.

### 2.10 Data analysis

Data are presented as geometric mean with standard deviation (SD) or number as a percent or median with interquartile range. The difference was considered statistically significant when p < 0.05. Statistical significance was determined when the p-value was less than 0.05. The statistical analysis was conducted using STATA-15, while the graphs were generated using GraphPad Prism 8.3.1.

## 3 Result

### 3.1 Detection of antibodies against the influenza virus

The RT-PCR of nasal swab samples from a cohort of 311 patients was performed to confirm their SARS-CoV-2 infection. Then we detected the presence of antibodies against Influenza strains. Plasma antibody titers against seasonal Influenza A (H1N1 and H3N2) and Influenza B were determined using the HAI assays. Among 311, 164 contained antibodies against all tested Influenza A (H1N1 and H3N2) and B type. All the samples have antibodies against Influenza B **(**Figure-01**)**. Only 16 samples were found that did not contain antibodies against both strains of Influenza A (H1N1 and H3N2).

**Fig. 1:**
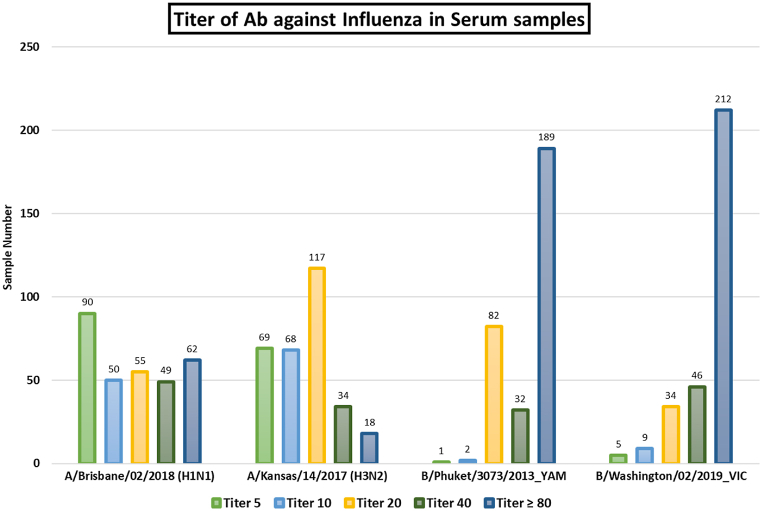
Titer of antibodies against the Influenza virus by Hemagglutination Inhibition (HAI) Assay among SARS-CoV-2 infected patients.

**Detection of presence and levels of SARS-CoV-2 S1 + S2 antibodies** SARS-CoV-2 S1 + S2 reactive S IgG was detected in samples, respectively. The corresponding cut-off was OD > 0.054 for the ELISA. SARS-CoV-2 S1 + S2 IgG was detected and had a range of antibody in the samples. Few do not even contain SARS-CoV-2 S1 + S2 antibodies. Based on antibody titer, samples were separated into three categories 208 (66.88 %) high (OD < 0.1), 47 (15.11 %) low (0.054<OD < 0.1), and 56 (18.01 %) absent (OD < 0.054) **(**Supplemenatary Figure-03**)**. Next, we determined the antibody titer of 16 specific samples (Influenza A negative) by using dilution ELISA. Among them, five samples have high (>2500), 7 have moderate (>1000), and 4 have low (<1000) affinity to bind with SARS-CoV-2 S1+S2 proteins **(**Figure-02**)**.

**Fig. 2:**
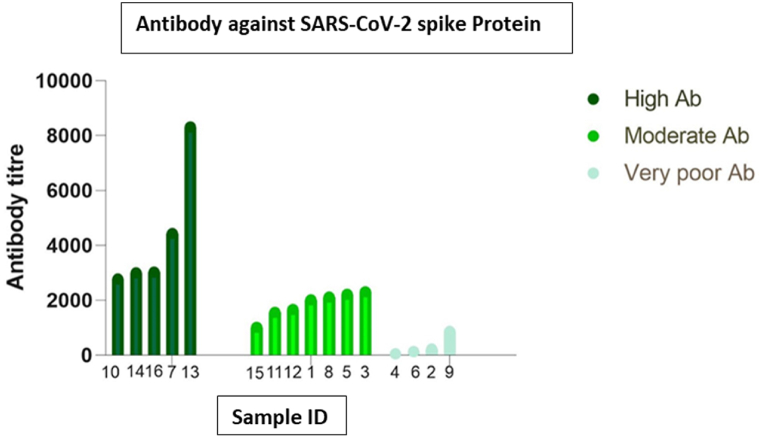
Detection of SARS-CoV-2 S-protein-specific antibody levels in study participants who do not have antibodies against the Influenza A virus.

**Fig. 3:**
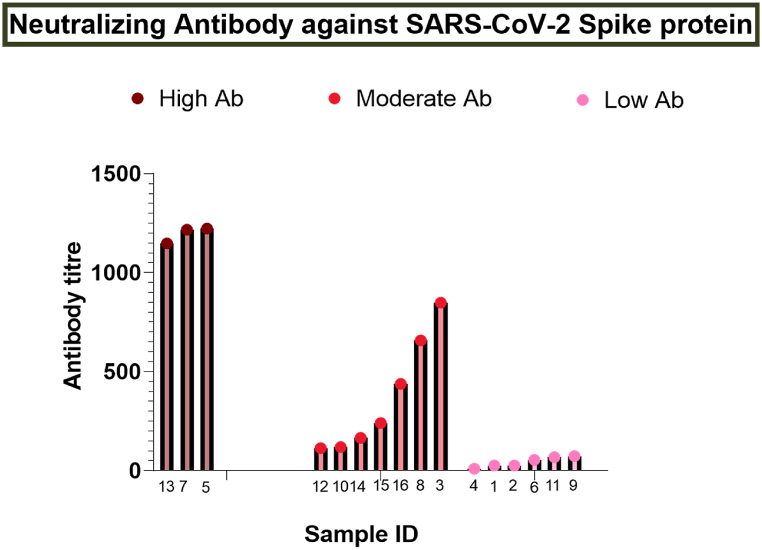
Representation of neutralization antibody titer against SARS-CoV-2 detected by Pseudo virus neutralization assay (PNA).

### 3.2 Determination of neutralizing antibodies against SARS-CoV-2

Samples with antibodies against SARS-CoV-2 were next screened for their neutralization capabilities against the SARS-CoV-2 Wuhan strain. Pseudo virus neutralization assay (PNA) was conducted to determine the neutralizing antibody titer. We found highly, moderately, and poorly neutralizing antibodies against SARS-CoV-2 among these 16 samples. The level of neutralizing antibodies (NT50) against SARS-CoV-2 was significantly higher (>1000) in three samples; seven had moderate (100–1000), and six samples (<100) had very poor neutralizing antibodies **(**Figure-03**)**.

### 3.3 Detection of influenza A virus neutralizing antibodies

In the Microneutralization (MN) assay, we assessed the plasma response using a standard Influenza virus dose. The reciprocal plasma dilution corresponding to the highest dilution with OD 490 less than 50 % of the cut-off (1.167) was considered the neutralization antibody titer for that plasma sample [38]. Among these 16 samples, 5 (33.33 %) samples (MN titer ≥20) have confirmed high, and 6 samples (37.5 %) (MN titer ≥10) have the moderate capability of neutralizing the Influenza A virus on MN assay **(**Figure-04**)**. 5 samples were not able to neutralize the Influenza virus. After sequencing, we found that all of the samples are delta variants that can neutralize Influenza A **(**Supplementary Table-01**)**.

**Fig. 4:**
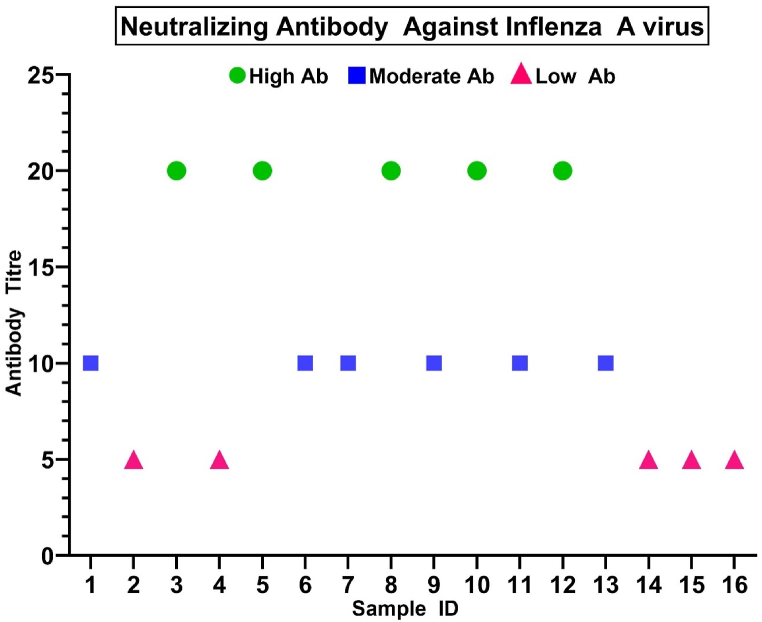
**Detection of the patient neutralizing Ab titer against Influenza A strains:** Here the blue circle dot, pink rectangle dot, and the green triangle dot represents samples that have high (MN titer ≥10) (MN titer ≥20), moderate (MN titer ≥10), and no (MN titer <10) neutralizing capability against Influenza A respectively.

### 3.4 Analysis for structural similarity

The interaction between Influenza A Hemagglutinin (HA) & Neuraminidase (NA) proteins and the antibody for the wild-type and delta variant spike protein of SARS-CoV-2 revealed distinct weighted scores and cluster sizes. The binding energy was calculated from the Prodigy server. The H1N1 HA protein showed the highest binding energy of −12.4 kcal/mol with a cluster size of 160, while H3N2 HA had a binding energy of −9.3 kcal/mol and a cluster size of 122 with antibody of delta variant spike protein **(**Table-01**)**. The H1N1 and H3N2 NA proteins exhibited binding energies of −10.1 and −11.7 kcal/mol, with cluster sizes of 61 and 86, respectively. That indicates a stronger binding affinity of the delta variant compared to the wild-type. Interestingly, the wild-type spike protein of SARS-CoV-2 had a binding energy of −11.1 kcal/mol with the antibody, and the largest cluster size of 224 **(**Table-01**)**, suggesting a strong interaction with the antibody, which is close to the energy of Influenza A proteins. The interactions between the proteins are visualized by ligPlot + v2.2 **(**Supplementary Figure-04**)**, which shows the presence of primarily hydrogens bonds and some hydrophobic bonds.

**Table 1:** Protein-Protein Docking result between Influenza A Hemagglutinin and Neuraminidase protein with the antibodies for wild-type and delta variant spike protein of SARS-CoV-2.

Name	Cluster Size	Binding Energy ΔG (kcal mol^−1^)
H1N1 HA vs. Delta Antibody	160	−12.4
H1N1 HA vs. wild type Antibody	81	−9.7
H3N2 HA vs. Delta Antibody	122	−9.3
H3N2 HA vs. wild type Antibody	43	−10.2
H1N1 NA vs. Delta Antibody	61	−10.1
H1N1 NA vs. wild type Antibody	63	−8.4
H3N2 NA vs. Delta Antibody	86	−11.7
H3N2 NA vs. wild type Antibody	80	−10.4
SARS-CoV-2 Spike vs. Delta Antibody	224	−11.1

In the comparative analysis of the structural similarity of Influenza-A, Hemagglutinin (HA) & Neuraminidase (NA) proteins with the wild-type spike protein of SARS-CoV-2, the TM-Score analysis was used. The HA protein of H1N1 and H3N2 proteins showed TM-Scores of 0.2997 and 0.2811, with aligned lengths of 111 and 102, respectively, with the spike protein. The NA protein of H1N1 and H3N2 proteins demonstrated slightly higher TM-Scores of 0.3273 and 0.3064, with aligned lengths of 127 and 121, respectively, with the spike protein **(**Supplementary Table-02**)**. This result indicates there is no structural similarity between spike protein and HA or NA protein of Influenza A. Interestingly, when comparing HA protein of H1N1 with H3N2, and NA protein of H1N1 with H3N2, the TM-Scores were significantly higher, at 0.8836 and 0.9578, respectively, indicating a higher level of structural similarity within the Influenza A subtypes **(**Supplementary Figure-05**).**

## 4 Discussion

Bangladesh is situated in the northern hemisphere and is characterized as a tropical country. The annual seasonal influenza epidemic normally takes place during the monsoon period, which spans from May to September [3]. On average, the peak activity of influenza lasts for around 12.5 weeks, starting from May (epi-weeks 18) and extending through July (epi-weeks 30.5) each year [9].

The influenza season in China during the period of 2019–2020 concluded at an earlier stage in comparison to previous years [39], and the United States and other Asian countries experienced a significant decrease in the prevalence of influenza [10,[40], [41], [42]].

In this study, we found a similar decline in seasonal influenza circulation [9]. And The temporal shift of the yearly seasonal influenza outbreak, typically observed between the months of May and September, has been observed to transpire from October to December in the year 2021, coinciding with the zenith of the COVID-19 pandemic [9].

Remarkably, there may be presence of coinfection between SARS-CoV-2 and influenza virus within the study groups under investigation, as depicted in Fig. 01 and Supplementary Figure 03. Among 311 SARS-CoV-2 positive samples, 94 % had antibodies against Influenza A and B strains that was confirmed by HAI assay. It depicts that flu were also circulaating during COVID-19 pandemics, but due to safety mesures and treatment adapted for SARS-CoV-2 help to protect individals fromflu. Several studies conducted on bigger cohorts have also observed a comparable increase in the percentage (52 %) of influenza virus infection among laboratory-confirmed cases infected with SARS-CoV-2. Additionally, in China and the USA, the respective rates of coinfection with other respiratory infections were reported to be 20 % [43,44].

Distinguishing between the clinical and laboratory features of Influenza and COVID-19 is a difficult job [45]. Zou et al. reported on two cases that were initially identified as Influenza but were afterwards confirmed to be COVID-19 [46]. Furthermore, there have been recent reports indicating the simultaneous transmission of both viruses during pandemics in several countries [47]. Diagnostic misclassification can provide significant challenges in these particular regions. The potential for cross-reactivity between SARS-CoV-2 and influenza viruses has the potential to impede precise clinical diagnosis and treatment, hinder the comprehension of underlying pathomechanisms, and complicate the creation of vaccines.

We cannot exclude the possibility that the phenomenon seen in this study is assay-specific false positive and not true antigenic cross-reactivity. In this study we gathered the some proofs to support our hypothesis of cross-reactivity between Influenza and SARS-CoV-2.

Our results demonstrating the cross-reactivity of samples from COVID-19 patients on Influenza, in addition to our in-silico protein structure analysis, suggests that at least some specific antigenic cross-reactivity exists (Table-01 & Supplementary figure-04). In addition, the Microneutralization assay (Figure-04), SARS-CoV-2 S1/S2-ELISA (Figure-02), and Pseudovirus neutralization assay (Figure-03) results strongly demonstrate the presence of cross-reactivity, possibly due to a significant antigenic similarity. Structural similarities between Influenza and SARS-CoV-2 may result in the selected epitope's identity. The target site for the SARS-CoV-2 neutralizing antibodies is spike protein receptor binding site (RBS). The hypervariable region in RBS with minimum footprints results in the breadth of neutralization that is intensified by the class switching property of immuniglobin G (IgG).

Moreover, SARS-S CoV-2's protein is highly glycosylated [48], similar to influenza hemagglutinin and neuraminidase [49] and other type I fusion proteins. The enveloped viruses; such as SARS-CoV-2 and influenza, often hijack host-cell glycosylation pathways and influence pathobiology and immune selection. These glycan motifs can lead to either immune evasion or viral neutralization by the production of cross-reactive antibodies that can lead to antibody-dependent enhancement (ADE) of infection [48,49], So, the cross reactivity may be happened due to glycan motifs of influenza and SARS-CoV-2 motif. It can also be a co-infection of both of this virus. Due to infrastructure and financial limitations, we didn't able to check the cross neutralization in polyclonal antibody level.

Therefore, it is only the use of assays that can detect antibodies specifically targeting particular epitopes that one can observe distinct and varied cross-reactivity. However, the Hemagglutination Assay (HA) and the SARS-CoV-2 ELISA are extensively employed on a global scale. Consequently, the matter of potential cross-reactivity associated with these assays holds significant consequences. Our research, along with that of other investigators, has demonstrated that this assay exhibits a remarkably high level of specificity, ranging from around 95 %–100 %. The sensitivity of the assay, however, varies depending on the duration of time that has elapsed before the onset of symptoms [50]. The hemagglutinin (HA) is widely utilized on a global scale for the purpose of diagnosing influenza due to its cost-effectiveness and accessibility for testing in resource-constrained environments. The prevalence of this virus in Southeast Asia and South America is comparable to that of most influenza infections, indicating its endemic nature in these regions.

Moreover, the presence of antigenic cross-reactivity between Influenza and COVID-19 gives rise to inquiries concerning the potential for shared protective immunity or exacerbation of the disease via antibody-dependent enhancement. This inquiry warrants further investigation in subsequent academic research. The cross-reactivity seen between particular SARS-CoV-2 and influenza protein structures may be attributed to their structural similarities. The findings of this study indicate that, irrespective of genetic divergence, the presence of similarities in protein structure may serve as a predictive factor for cross-reactivity among viruses originating from phylogenetically different lineages. This work presents findings on the induction of Influenza cross-reactive SARS-CoV-2 S1-RBD specific antibodies through immunization with the SARS-CoV-2 delta variant. During the computational examination of structural similarities, it was observed that the receptor-binding domain (RBD) of SARS-CoV-2 does not exhibit any noteworthy or consistent similarities with the hemagglutinin (HA) and neuraminidase (NA) proteins. Nevertheless, our protein-protein docking analysis revealed that the binding energy associated with the interaction between the hemagglutinin (HA) and neuraminidase (NA) proteins of Influenza, and the antibody targeting SARS-CoV-2, has a comparable strength to the bond formed between the spike protein of SARS-CoV-2 and its corresponding antibody.

The findings presented in this study provide further evidence in support of the observed phenomenon in our experimental investigations with anti-S1-RBD IgG derived from rabbit serum. Additionally, these data provide credence to the hypothesis we put up, which suggests that anti-S1-RBD antibodies have the potential to impede influenza infection through cross-reactivity with influenza antigens. Nevertheless, it is important to acknowledge that our sample size is limited, and thus, we cannot entirely dismiss the potential for these COVID-19 individuals to have been previously or currently infected with Influenza. However, concurrently, no anti-influenza antibodies were detected among the patients. Hence, additional research is required to validate the potential of anti-SARS-COV-2 antibodies in impeding Influenza infection. In this study, we have successfully shown that there exists an antigenic resemblance between the S1-RBD of SARS-CoV-2 and Influenza. Consequently, there is a possibility of generating Influenza cross-reactive antibodies through S1-RBD immunization or infection with SARS-CoV-2.

To conclude, concurrent detailed and large-scale studies are necessary to investigate Cross-neutralization of Influenza A by SARS-CoV-2. More studies focusing on the functional relevance of this viruses are needed, as well as more thorough investigations to determine which specific protein is responsible for this process.

## CRediT authorship contribution statement

**Mohammad Mamun Alam:** Writing – review & editing, Supervision, Project administration, Investigation, Funding acquisition, Data curation. **Asma Salauddin:** Writing – review & editing, Writing – original draft, Project administration, Methodology, Data curation, Conceptualization. **Sayra Moni:** Project administration, Methodology. **Md Belayet Hasan Limon:** Writing – original draft, Methodology. **Raisha Musarrat:** Methodology. **Sagar Bosu:** Methodology. **Mohammad Enayet Hossain:** Data curation. **Mohammed Ziaur Rahman:** Funding acquisition. **Mustafizur Rahman:** Writing – review & editing, Funding acquisition, Data curation.

## Research data for this article

Due to the sensitive nature of the questions asked in this study, survey respondents were assured raw data would remain confidential and would not be shared. The data that has been used is confidential.

## Declaration of competing interest

The authors declare that they have no known competing financial interests or personal relationships that could have appeared to influence the work reported in this paper.
