# Supplementary material for: Cross-neutralization of Influenza A by SARS-CoV-2 specific neutralizing antibodies and polyclonal plasma: Is pre-exposure to SARS-CoV-2 protective against Influenza A?
Source: Heliyon. 2024 Nov 22;10(23):e40638. doi: 10.1016/j.heliyon.2024.e40638 (PMC11626022; doi:10.1016/j.heliyon.2024.e40638)
Supplement: Multimedia component 1 [file mmc1.docx]

**Supplementary Data**

| Sample Dilution | Virus Control (VC) | NC | | | 156 | | | 165 | | | Cell Control (CC) | |
| --- | --- | --- | --- | --- | --- | --- | --- | --- | --- | --- | --- | --- |
| 1 | 1793062 | 1696840 | 1483158 | 1476509 | 43384 | 26583 | 39757 | 88376 | 190249 | 133285 | 1152 |  |
| 2 | 1788522 | 1624737 | 1421442 | 1533507 | 138372 | 105264 | 155608 | 100526 | 374202 | 318532 | 2258 |  |
| 4 | 1450603 | 1622137 | 1496419 | 1614060 | 606851 | 427930 | 338702 | 542043 | 723009 | 695190 | 1587 |  |
| 8 | 1663501 | 1686373 | 1541441 | 1622264 | 751974 | 634359 | 605980 | 1140997 | 1074719 | 1004060 | 2127 |  |
| 16 | 1773860 | 1659069 | 1629902 | 1670194 | 1099788 | 981930 | 782727 | 1245551 | 1175908 | 1332438 | 2976 |  |
| 32 | 1622131 | 1690576 | 1663899 | 1692560 | 1313237 | 1203115 | 1120570 | 1502857 | 1308698 | 1382214 | 3540 |  |

**Supplementary Figure-01:** Plate map of the PNA and Graph of the Neutralization Assay.

|  | S1 | S2 | S3 | S4 | S5 | S6 | S7 | S8 | S9 | NC | Back Titre | 12 |  |
| --- | --- | --- | --- | --- | --- | --- | --- | --- | --- | --- | --- | --- | --- |
| A (1:10) | 0.69 | 2.251 | 0.169 | 0.202 | 0.952 | 0.861 | 0.189 | 0.693 | 0.323 | 1.730 | 2.451 | 2.199 | VC |
| B (1:20) | 1.713 | 2.102 | 0.317 | 1.149 | 1.874 | 1.729 | 0.805 | 1.384 | 1.045 | 2.123 | 2.319 | 1.948 |  |
| C (1:40) | 2.17 | 2.206 | 1.615 | 2.008 | 2.142 | 2.057 | 1.839 | 1.986 | 1.953 | 2.132 | 2.734 | 2.286 |  |
| D (1:80) | 2.231 | 2.242 | 2.01 | 2.075 | 2.147 | 2.040 | 2.008 | 2.067 | 2.098 | 2.118 | 2.535 | 2.364 |  |
| E (1:160) | 2.276 | 2.233 | 2.147 | 2.080 | 2.127 | 2.003 | 2.043 | 2.085 | 2.104 | 2.093 | 2.524 | 0.090 | CC |
| F (1:320) | 2.175 | 2.109 | 2.238 | 2.145 | 2.186 | 2.104 | 2.085 | 2.127 | 2.147 | 2.150 | 2.420 | 0.103 |  |
| G (1:640) | 2.176 | 2.089 | 2.112 | 2.198 | 2.413 | 2.040 | 2.101 | 2.135 | 2.109 | 1.978 | 2.260 | 0.079 |  |
| H (1:1280) | 2.183 | 2.153 | 2.305 | 2.279 | 2.249 | 2.153 | 2.148 | 2.172 | 2.106 | 2.198 | 2.291 | 0.093 |  |

**Supplementary Figure-02:** Plate map of the Microneutralization Assay.

**Supplementary Figure-03:** Detection of presence and distribution of SARS-CoV-2 S1/S2 antibodies among the cohort.

**Supplementary Table- 01:** Detection of SARS-CoV-2 variant among patients with SARS-CoV-2 neutralizing antibodies positive and Influenza A neutralizing antibodies negative

| **Sample ID** | **Variant** | **Pangolineage and Accession Number** |
| --- | --- | --- |
| 1 | Delta | B.1.617.2 (Pango v.4.2 PLEARN-v1.18), Delta (B.1.617.2-like) (Scorpio), Sequence not Uploaded |
| 2 | Eta | B.1.525 (Pango v.4.2 PLEARN-v1.18), Eta (B.1.525-like) (Scorpio), EPI_ISL_11324408 |
| 3 | Delta | B.1.617.2 (Pango v.4.2 PLEARN-v1.18), Delta (B.1.617.2-like) (Scorpio), EPI_ISL_11324530 |
| 4 | Delta | B.1.617.2 (Pango v.4.2 PLEARN-v1.18), Delta (B.1.617.2-like) (Scorpio), EPI_ISL_11324421 |
| 5 | Delta | B.1.617.2 (Pango v.4.2 PLEARN-v1.18), Delta (B.1.617.2-like) (Scorpio), EPI_ISL_11324498 |
| 6 | Delta | B.1.617.2 (Pango v.4.2 PLEARN-v1.18), Delta (B.1.617.2-like) (Scorpio), EPI_ISL_11324495 |
| 7 | Delta | B.1.617.2 (Pango v.4.2 PLEARN-v1.18), Delta (B.1.617.2-like) (Scorpio), EPI_ISL_11324499 |
| 8 | Delta | B.1.617.2 (Pango v.4.2 PLEARN-v1.18), Delta (B.1.617.2-like) (Scorpio), EPI_ISL_11324496 |
| 9 | Delta | B.1.617.2 (Pango v.4.2 PLEARN-v1.18), Delta (B.1.617.2-like) (Scorpio), EPI_ISL_11324513 |
| 10 | Delta | B.1.617.2 (Pango v.4.2 PLEARN-v1.18), Delta (B.1.617.2-like) (Scorpio), EPI_ISL_11324522 |
| 11 | Delta | B.1.617.2 (Pango v.4.2 PLEARN-v1.18), Delta (B.1.617.2-like) (Scorpio), EPI_ISL_11324511 |
| 12 | Delta | B.1.617.2 (Pango v.4.2 PLEARN-v1.18), Delta (B.1.617.2-like) (Scorpio), EPI_ISL_11324536 |
| 13 | Delta | B.1.617.2 (Pango v.4.2 PLEARN-v1.18), Delta (B.1.617.2-like) (Scorpio), EPI_ISL_11324519 |
| 14 | Delta | B.1.617.2 (Pango v.4.2 PLEARN-v1.18), Delta (B.1.617.2-like) (Scorpio), EPI_ISL_11324576 |
| 15 | N/A | B.1.1.25 (Pango v.4.2 PLEARN-v1.18), EPI_ISL_13477155 |
| 16 | N/A | B.1.1.25 (Pango v.4.2 PLEARN-v1.18), EPI_ISL_12845601 |


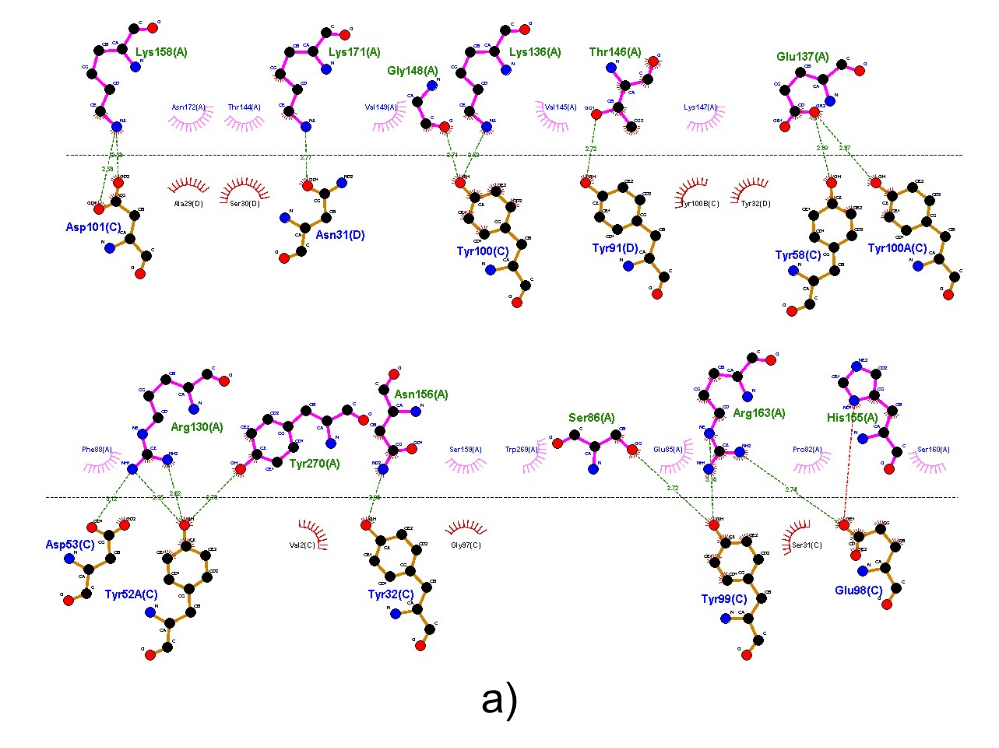

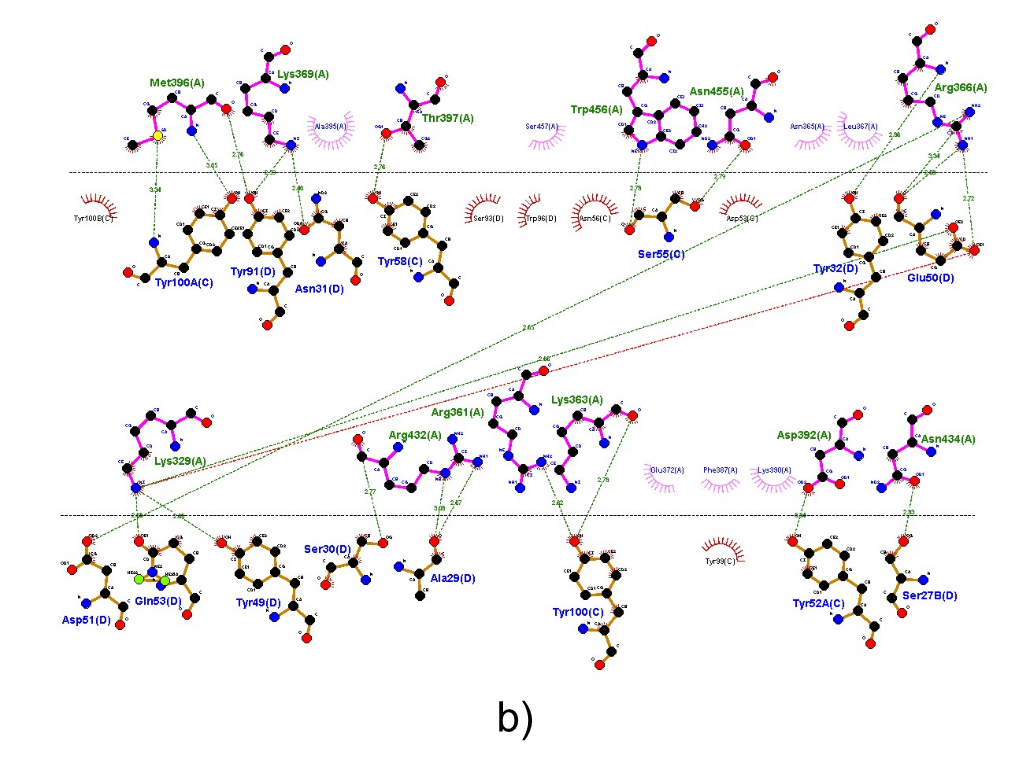


Hydrogen bonds

Hydrophobic bonds


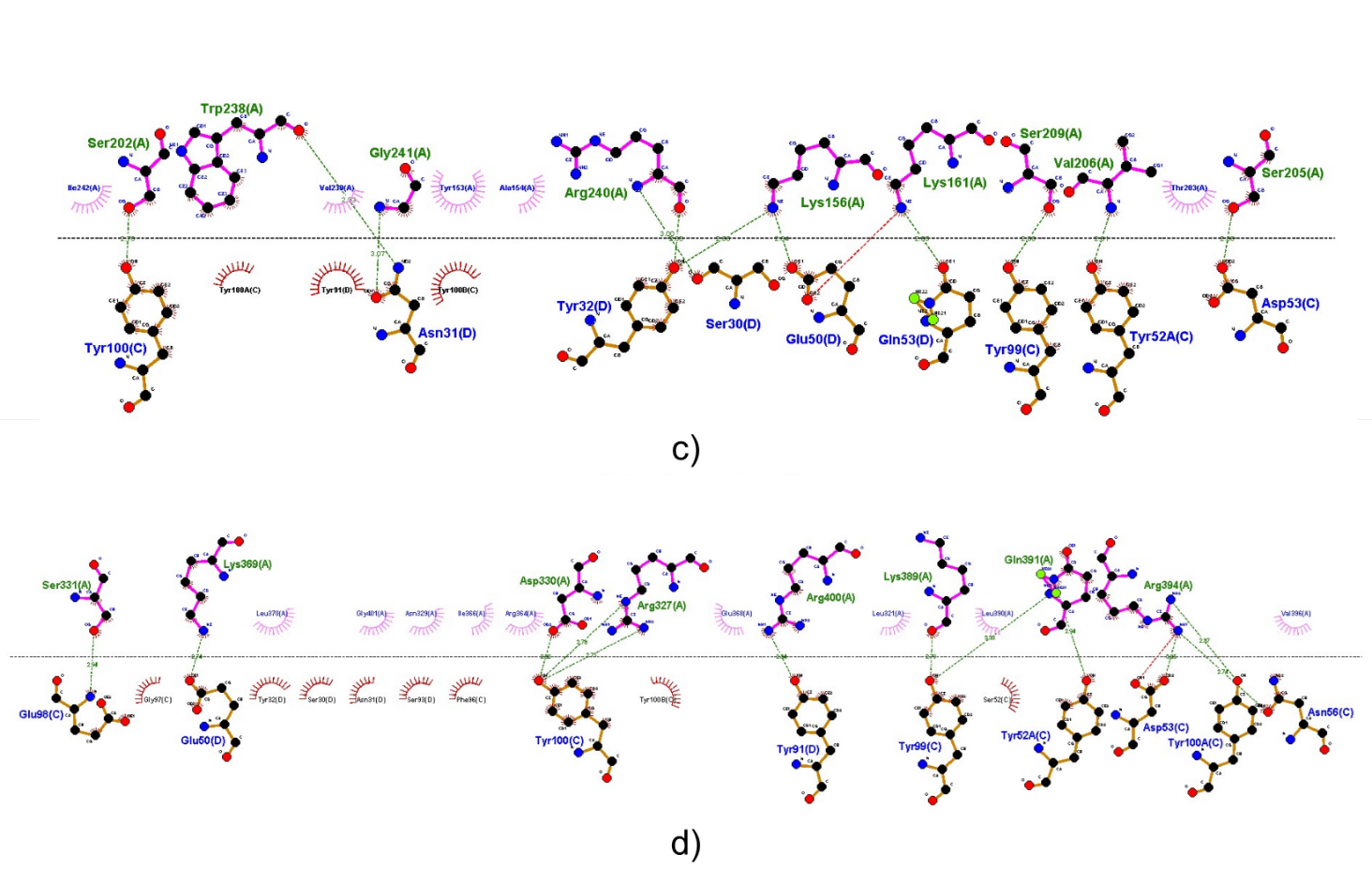


**Supplementary Figure- 04:** Influenza A HA and NA Protein-Protein docking with Antibody for Spike protein of SARS-CoV-2: a) HA of H1N1 and Antibody for delta variant Spike protein; b) NA of H1N1 and Antibody for delta variant Spike protein; c) HA of H3N2 and Antibody for delta variant Spike protein; d) HA of H3N2 and Antibody for delta variant Spike protein

**Supplementary Table-02:** TM-Score Result between Influenza A Hemagglutinin & Neuraminidase protein and wild-type spike protein of SARS-CoV-2.

| **Name** | **TM-Score** | **Aligned Length** |
| --- | --- | --- |
| HA H1N1 vs. Delta Spike | 0.2997 | 111 |
| HA H3N2 vs. Delta Spike | 0.2811 | 102 |
| NA H1N1 vs Delta Spike | 0.3273 | 127 |
| NA H3N2 vs Delta Spike | 0.3064 | 121 |
| HA H1N1 vs H3N2 | 0.8836 | 483 |
| NA H1N1 vs H3N2 | 0.9578 | 383 |


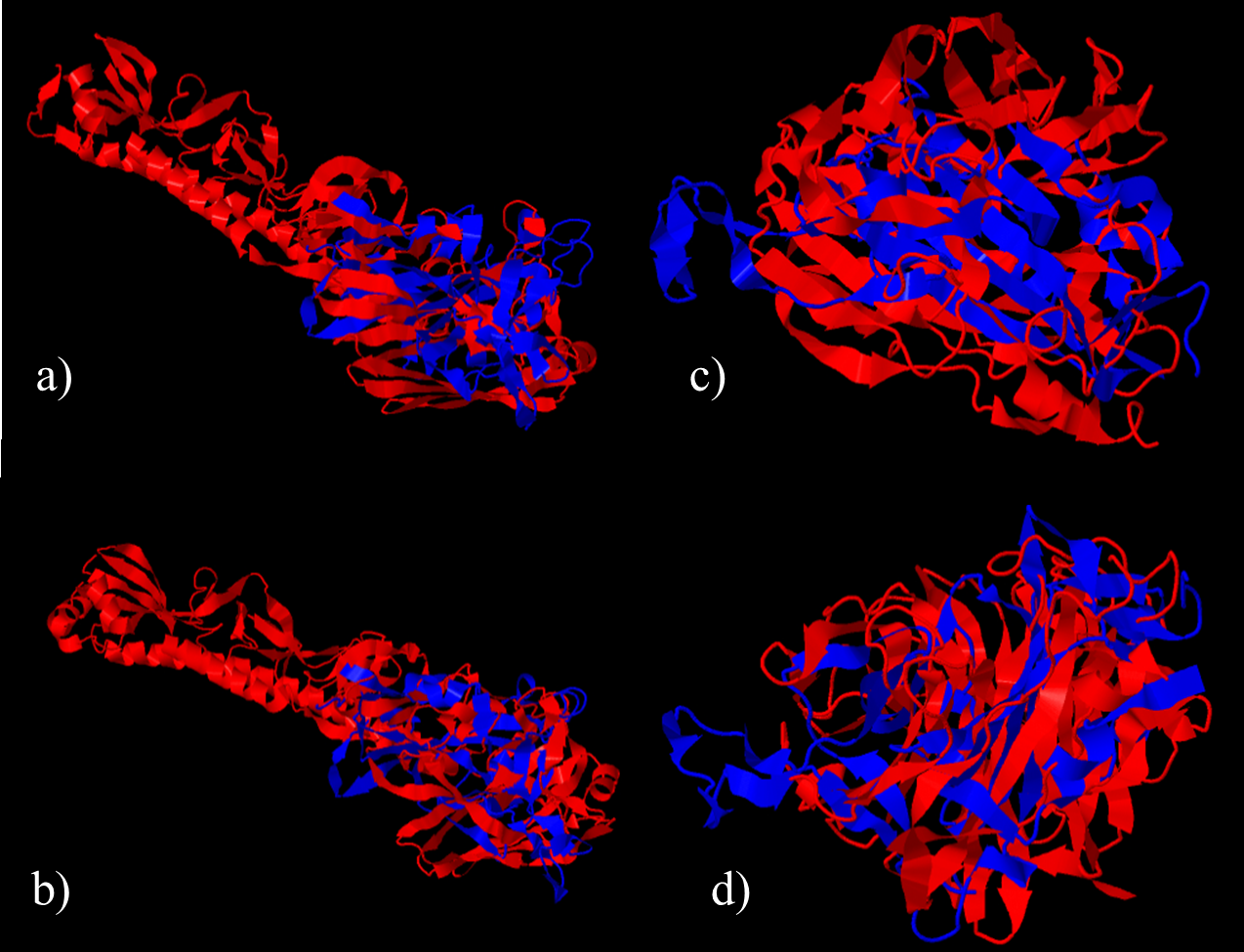


**Supplementary Figure- 05:** Structural Alignment by TM-Align tool: Red color denotes the HA and NA protein of Influenza A virus, and Blue color denotes the antibody for delta variant spike protein of SARS-CoV-2; Here, a) HA protein of H1N1 aligned with SARS-CoV-2 Spike protein; b) HA protein of H3N2 aligned with SARS-CoV-2 Spike protein; c) NA protein of H1N1 aligned with SARS-CoV-2 Spike protein; d) NA protein of H3N2 aligned with SARS-CoV-2 Spike protein.
